# Risk factors and new diagnostic index for deep venous thrombosis of lower extremities in elderly patients with traumatic femoral neck fracture

**DOI:** 10.3389/fsurg.2022.1050347

**Published:** 2023-02-21

**Authors:** Wenhao Chen, Zhiming Su, Quan Liu, Xinxin Bai, Jiyue Huang, Shaohuang Weng, Min Chen

**Affiliations:** ^1^ Department of Orthopedic Surgery, Fujian Medical University Union Hospital, Fuzhou, China; ^2^ Department of Pharmaceutical Analysis, School of Pharmacy, Fujian Medical University, Fuzhou, China

**Keywords:** femoral neck fracture, deep venous thrombosis, predictor, risk factors, elderly

## Abstract

**Purpose:**

To determine the incidence and risk factors of deep vein thrombosis (DVT) of lower extremities at admission in elderly Chinese patients with femoral neck fracture, and to establish and evaluate a new DVT predictor based on these risk factors.

**Methods:**

Patients who were hospitalized from January 2018 to December 2020 at three independent centers were reviewed. According to the results of lower extremities vascular ultrasound at admission, they were divided into DVT group and non-DVT group. Single and multivariate logistic regression analysis were applied to identify independent risk factors for DVT occurrence, and then a prediction formula for DVT based on the risk factors was developed. The new predictive index of DVT was calculated by the formula. Receiver operating characteristic (ROC) curve analysis was used to determine the diagnostic value of different factors and the new predictive index.

**Results:**

There were 203 elder patients were included in the final analysis after application of the exclusion criteria. Thirty seven patients (18.2%) were diagnosed as DVT by ultrasound, including 33 patients (89.2%) of peripheral type, 1 patient (2.7%) of central type and 3 patients (8.1%) of mixed type.Multivariate logistic regression analysis showed that four factors including injured side, hemoglobin, fibrinogen, d-dimer were the independent risk factors for the incidence of DVT. On this basis, a new formula for DVT predictive factor was constructed: New predictive index = 0.895 * injured side (right = 1, left = 0) + 0.899 * hemoglobin (<109.5 g/L = 1, > 109.5 g/L = 0) + 1.19 * fibrinogen (>4.24 g/L = 1, < 4.24 g/L = 0) + 1.221* d-dimer (>2.4 mg/L = 1, < 2.4 mg/L = 0). The AUC value of this new developed index was 0.735.

**Conclusions:**

This work showed that the incidence of DVT in elderly patients with femoral neck fracture in China was high at admission. New DVT predictive value can be used as an effective diagnosis strategy for evaluation of thrombosis at admission.

## Introduction

Due to the increased bone fragility and impaired walking ability in the elderly (1), femoral neck fractures are becoming more common (2). With the aging of the population, the number of elderly patients with femoral neck fractures caused by trauma who need surgery has increased significantly (3). Lower extremities deep venous thrombosis is one of the common complications of lower extremity trauma. Dislodgment of thrombus can lead to pulmonary embolism (PE), which is the third most common cause of death in patients who survive within 24 h after trauma (4). DVT or PE is one of the most serious complications in patients with femoral neck fracture, which can increase the perioperative mortality (5, 6). Early operation in patients with femoral neck fractures can reduce the risk of perioperative complications and death (8). However, we had to extend the patient's preoperative waiting time to conduct anticoagulation therapies if the patient had DVT of lower extremities. The diagnosis of DVT is mainly through plasma markers, vascular ultrasound and venography (9). Although venography is the gold standard for the diagnosis of venous thromboembolism (10), its application is limited in clinical work ascribed to the invasiveness and unrepeatability. In recent years, ultrasonography has become more and more popular in clinic (11) because of the characteristic of non-invasiveness and repeatable examination. However, ultrasound examination needs to adjust the body position, which is not suitable for all patients with fracture, especially those with hip neck fracture. Moreover, the accuracy of ultrasonography is also affected by the professional knowledge of the executive physician.

As the final product of plasmin mediated degradation of cross-linked fibrin, d-dimer is a plasma marker with certain significance for patients with suspected DVT (12). However, the low specificity of d-dimer induced the dispute of the diagnostic value of D-dimer in elderly patients. Recently, some studies suggested that D-dimer and age were the risk factors of hip fracture and lower extremities venous thrombosis, and established a prediction model (7). However, the prediction model was fabricated based on the patients with femoral neck fracture and femoral intertrochanteric fracture. It has reported that intertrochanteric fracture itself is a risk factor for DVT of lower extremities (4). And the risk of DVT in patients with intertrochanteric fracture is 2.5 times than that of femoral neck fracture (4). Therefore, it is of great clinical significance to clarify the incidence and risk factors of lower extremities deep venous thrombosis in patients with femoral neck fracture. Furthermore, the fabrication of a predictive method based on risk factors for DVT for the preoperative diagnosis of DVT of lower extremities in patients with femoral neck fractures is significant for clinical management of thrombotic patients.

This study aimed to identify the incidence and risk factors of lower extremities DVT in elderly patients with femoral neck fractures in China, and to establish and evaluate a new index of DVT based on the risk factors to help clinicians reduce the misdiagnosis rate of DVT.

## Materials and methods

**Figure 1:**
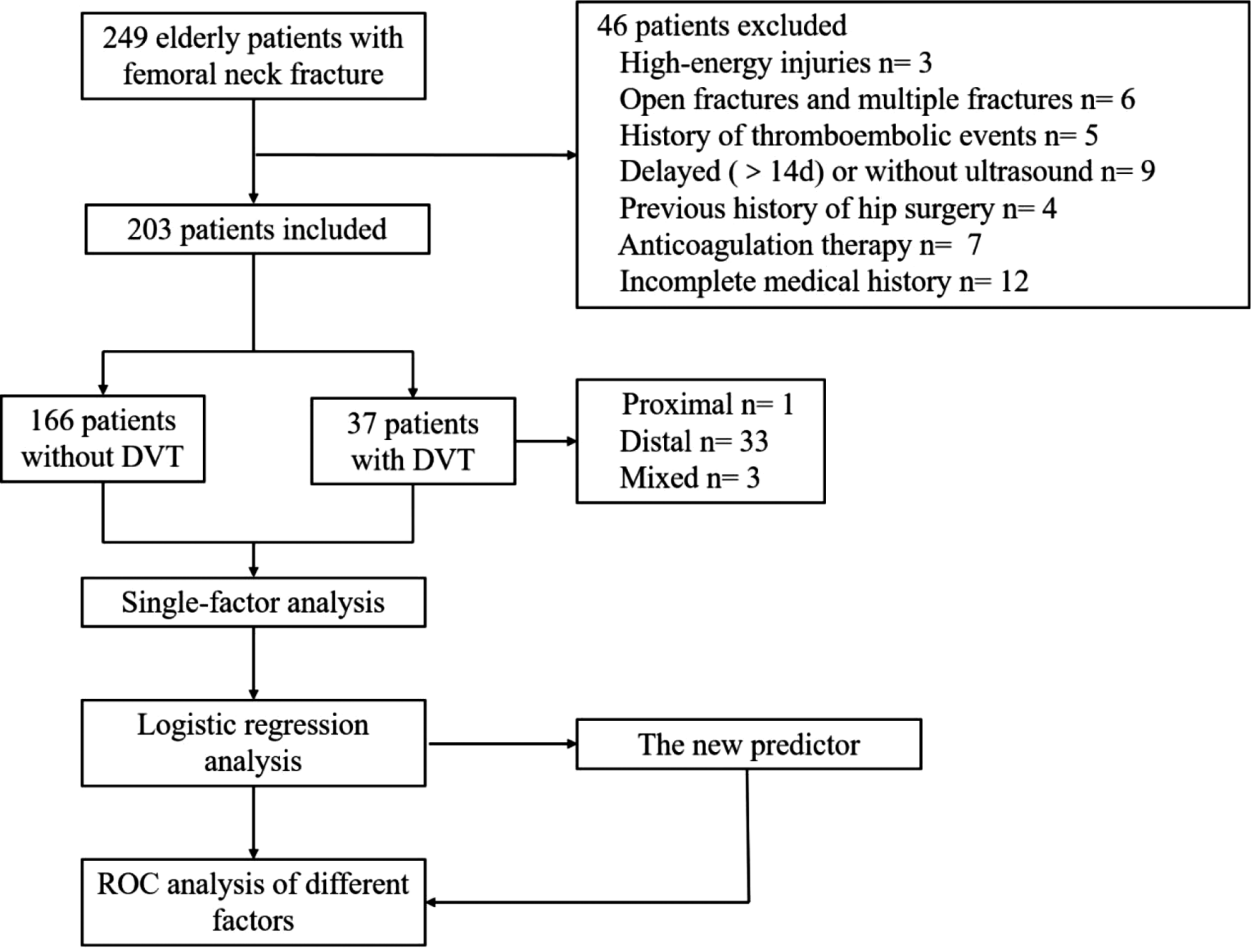
The sampling procedure used for all elderly patients with femoral neck fracture in this study.

This study was a retrospective survey conducted at three medical centers. We retrospectively reviewed all elderly patients with femoral neck fractures identified by hip x-ray and/or 3D CT scans at Fujian Medical University Union Hospital, Fujian Provincial Hospital, and Fuzhou General Hospital. Inclusive criteria: unilateral femoral neck fractures and closed fractures caused by low energy trauma (falling from standing height) of the patients aged 60 or above. The exclusion criteria were high-energy injuries, open fractures, multiple fractures, history of thromboembolic events, delayed or without ultrasound (more than 14 days after fracture of the lower extremity), previous history of hip surgery, anticoagulation therapy (such as aspirin, heparin, and low-molecular-weight heparin) 7 days before fracture, and incomplete medical history. The sampling procedure of all the elderly patients with femoral neck fractures in this study was shown in Figure 1.

Inpatient record systems are used to retrieve patient data, including demographics (age, sex, side of injury, time from injury to ultrasound), medical history (cerebrovascular disease, malignant tumor), comorbidities (including hypertension, diabetes, arrhythmias, renal insufficiency, Parkinson's disease and coronary heart disease), laboratory tests (neutrophils count, lymphocyte count, hemoglobin, platelets, albumin, cholesterol, HDL, fibrinogen and d-dimer). According to the Rabinov criteria (14), DVTs of lower extremities are divided into central, peripheral and mixed types. DVTs of lower extremities involving popliteal vein and/or deep vein above popliteal vein (superficial femoral vein, deep femoral vein, and common femoral vein) are central DVTs. Peripheral DVTs occur when thrombus occur in anterior and/or posterior tibial veinand/or peroneal vein and/or muscular calf veins of the leg. Both of them are mixed. The patients were divided into DVT group and non-DVT group according to the results of bilateral lower limb vascular Doppler ultrasonography at admission.

### Statistical analysis

All analyses were performed using SPSS 25.0 (SPSS, IBM, Armonk, New York, United States). Continuous variables are represented by mean and standard deviation (SD), and evaluated by *T* test. Categorical variables are expressed as proportion (%), using *χ*^2^ test or Fisher exact test to assess. Considering the significance and simplified calculation in clinical practice, the receiver operating characteristic (ROC) curve was used to determine the optimal critical value of quantitative data. Single logistic regression analysis was used to determine which independent variables had significant differences between the two groups. Variables tested as approximately significant (*P* < 0.05) in univariate analysis were included in multivariate models to identify independent risk factors for DVT occurrence and the coefficient regression (CR) of the related independent variables. The predictive index of DVT is calculated by coefficient regression and independent variable formula. The best critical value of quantitative data, the sensitivity and specificity of quantitative data and the diagnostic value of relevant factors were determined by using receiver operating characteristic curve (ROC). The statistical test level is set to *P* < 0.05.

## Results

A total of 203 elderly Chinese patients with femoral neck fractures (66 males and 137 females) were included in this study. Based on the results of bilateral lower extremities vascular ultrasound, they were divided into two groups: DVT group and none DVT. The demographics of the patients were shown in Table 1. In the study, 37 patients were diagnosed with DVTs of lower extremities by ultrasound. The admission incidence of DVT was 18.2%. Of these 37 patients with DVT, 33 (89.2%) had peripheral DVTs, 1 (2.7%) had central DVTs, and 3 (8.1%) had mixed DVTs.

**Table 1:** Comparison between with DVT and without DVT patients with femoral neck fractures.

Items	DVT group (*n* = 37)	Non-DVT group (*n* = 166)	Overall (*n* = 203)	*P*-value
Gender (female)	29 (78.4%)	108 (65.1%)	137 (67.5%)	0.118
Age (>72.5year)	28 (75.7%)	92 (55.4%)	120 (59.1%)	0.023
Injured side (right)	23 (62.2%)	71 (42.8%)	94 (46.3%)	0.032
Diabetes	9 (24.3%)	45 (27.1%)	54 (26.6%)	0.729
Hypertension	23 (62.2%)	97 (58.4%)	120 (59.1%)	0.677
History of malignancy	7 (18.9%)	22 (13.3%)	29 (14.3%)	0.373
Parkinson's disease	1 (2.7%)	9 (5.4%)	10 (4.9%)	0.786
Renal dysfunction	3 (8.1%)	7 (4.2%)	10 (4.9%)	0.569
Coronary heart disease	4 (10.1%)	38 (22.9%)	42 (20.7%)	0.101
Arrhythmia	4 (10.1%)	28 (16.9%)	32 (15.8%)	0.361
Chronic heart disease	1 (2.7%)	19 (11.4%)	20 (9.9%)	0.107
Cerebrovascular disease	4 (10.1%)	27 (16.3%)	31 (15.3%)	0.404
Time from injury to ultrasound (days)	4.3 ± 3.6	3.5 ± 2.9	3.6 ± 3.1	0.144
HB (<109.5 g/L)	14 (37.8%)	27 (16.3%)	41 (20.2%)	0.003
PLT (<145.5 × 10^9^/L)	25 (67.6%)	139 (83.7%)	164 (80.8%)	0.024
TC (>4.665 mmol/L)	18 (48.6%)	70 (42.2%)	88 (43.3%)	0.472
HDL (<1.225 mmol/L)	11 (29.7%)	76 (45.8%)	87 (42.9%)	0.074
ALB (<36.85 g/L)	26 (70.3%)	93 (56.0%)	119 (58.6%)	0.112
Fibrinogen (>4.24 g/L)	22 (59.5%)	66 (39.8%)	88 (43.3%)	0.029
D-dimer (>2.4 mg/L)	30 (81.1%)	106 (63.9%)	136 (67.0%)	0.044
NEU (<5.545 × 10^9^/L)	16 (43.2%)	54 (32.5%)	70 (34.5%)	0.215
LYM (<0.64 × 10^9^/L)	6 (16.2%)	11 (6.6%)	17 (8.4%)	0.115

DVT, deep vein thrombosis; HB, hemoglobin; PLT, platelets; TC, total cholesterol; HDL, high-density lipoprotein; ALB, serum albumin; NEU, neutrophils; LYM, lymphocyte.

In univariate analysis, injury side (right), hemoglobin (<109.5 g/L), platelet (<145.5 × 109), fibrinogen (<4.24 g/L), d-dimer (>2.4 mg/L), and age (>72) were significantly associated with DVT (*P* < 0.05) in elderly patients with femoral neck fractures (Table 2). Multivariate logistic regression analysis (Table 3) confirmed that the 4 significant risk factors for DVT in elderly patients with femoral neck fractures were: injured side (right) (OR: 2.448, *P* = 0.029), hemoglobin (<109.5 g/L) (OR: 2.457 *P* = 0.044), fibrinogen (>4.24 g/L) (OR: 3.288, *P* = 0.009), and d-dimer (>2.4 mg/L) (OR: 3.391, *P* = 0.017). Therefore, injured side (right, OR: 2.448), hemoglobin (<109.5, OR: 2.457), fibrinogen (>4.24 g/L, OR: 3.288), and d-dimer (>2.4 mg/L, OR: 3.391) can be used to predict the occurrence of DVT at admission in elderly patients with femoral neck fractures in China. The new DVT predictive index is calculated according to the following formula: new predictive index = 0.895 * injured side (right) + 0.899 * hemoglobin (<109.5 g/L) + 1.19 * fibrinogen (>4.24 g/L) + 1.221 * d-dimer (> 2.4 mg/L).

**Table 2:** Univariate analysis of admission DVT in elderly patients with femoral neck fracture.

Risk Factors	OR	95% CI	*P*-value
Injured side (right)	2.198	1.057–4.571	0.035
HB (<109.5 g/L)	3.134	1.434–6.849	0.004
PLT (<145.5 × 10^9^/L)	2.471	1.108–5.512	0.027
Fibrinogen (>4.24 g/L)	2.521	1.048–6.061	0.031
D-dimer (>2.4 mg/L)	2.545	1.189–5.448	0.049
Age (>72.5year)	2.443	1.071–5.573	0.027

OR, odds ratio; 95% CI, 95% confidence interval.

**Table 3:** Multivariate analysis of admission DVT in elderly patients with femoral neck fracture.

Risk Factors	CR	SE	Wald	OR	95% CI	*P*-value
Injured side (right)	0.895	0.409	4.789	2.448	1.098–5.457	0.029
HB (<109.5 g/L)	0.899	0.445	4.075	2.457	1.026–5.883	0.044
Fibrinogen (>4.24 g/L)	1.190	0.456	6.817	3.288	1.346–8.036	0.009
D-dimer (>2.4 mg/L)	1.221	0.512	5.684	3.391	1.243–9.252	0.017

CR, coefficient regression; SE, standard error; OR, odds ratio; 95% CI, 95% confidence interval.

**Figure 2:**
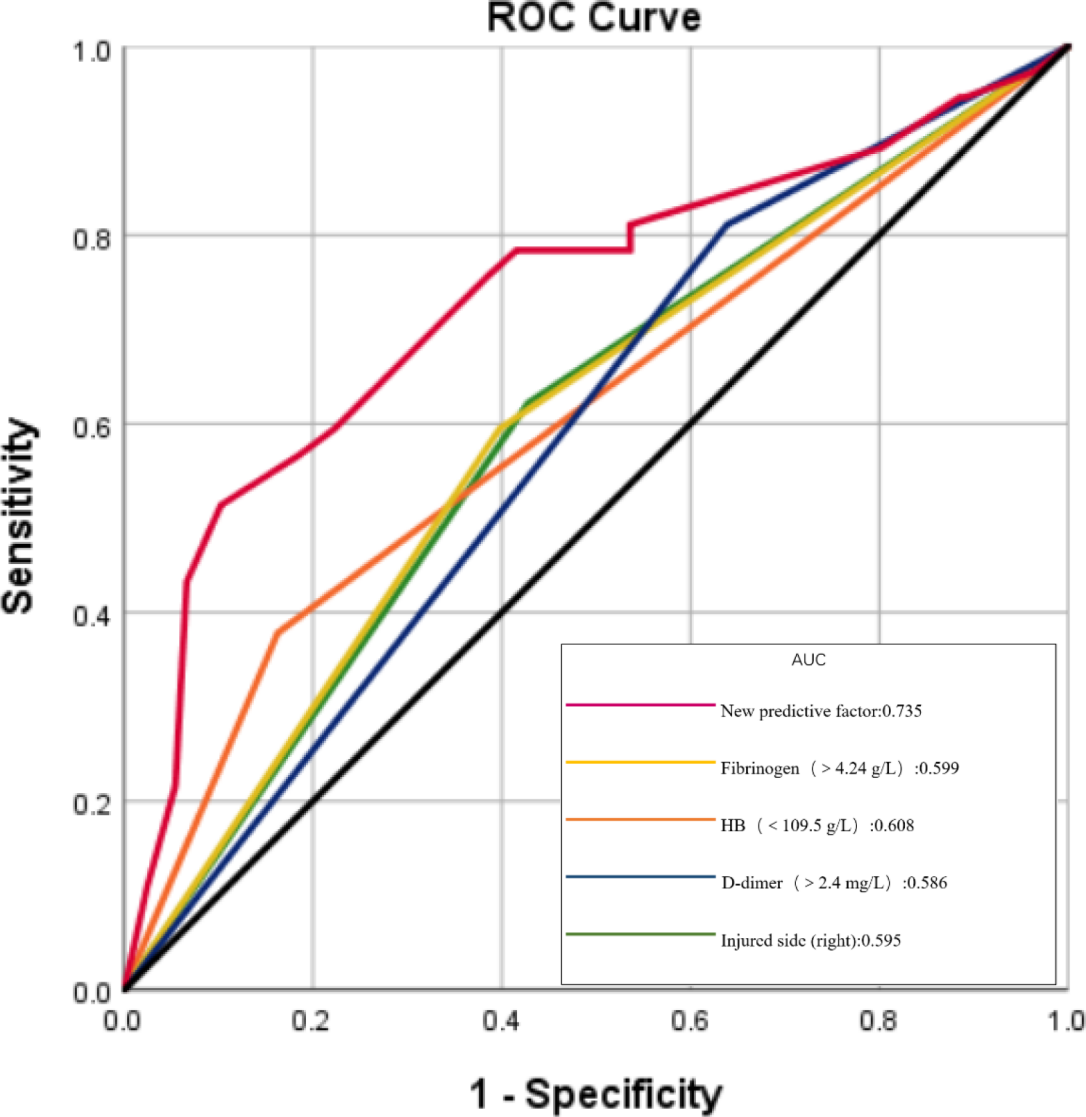
ROC curves for the diagnosis of DVTs based on AUC (the area under the curve).

According to the developed predictive formula, the new predictive value of DVT group (2.59 ± 1.04) was higher than that of non-DVT group (1.78 ± 0.81) with significant difference. ROC curve was used to analyze the diagnostic value of these risk factors. The ROC curves of each factor were shown in Figure 2. The value of the area under the ROC curves (AUC) indicate the diagnostic value of the predictors. The AUC value of the new DVT predictive index was 0.735, which was highest among the factors. Youden index was used to calculate the diagnostic threshold of these predictors. The diagnostic threshold of the new predictor was 2.70, the sensitivity was 51.4%, and the specificity was 89.8%. The sensitivities of injured side (right) (OR: 2.448), hemoglobin (<109.5 g/L) (OR: 2.457), fibrinogen (>4.24 g/L) (OR: 3.288), and d-dimer (>2.4 mg/L) (OR: 3.391) were 62.2%, 37.8%, 59.5% and 81.1% respectively; the specificity was 57.2%, 83.7%, 60.2% and 36.1% respectively; and the AUC value was 0.597, 0.608, 0.599 and 0.586 respectively. (Table 4).

**Table 4:** The ROC results of different factors.

Risk Factors	Sensitivity	Specificity	AUC
Injured side (right)	62.2%	57.2%	0.595
HB (<109.5 g/L)	37.8%	83.7%	0.608
Fibrinogen (>4.24 g/L)	59.5%	60.2%	0.599
D-dimer (>2.4 mg/L)	81.1%	36.1%	0.586
New predictive factor	51.4%	89.8%	0.735

AUC, the area under the curve.

## Discussion

Among the patients with hip fractures, 8%–34.9% had preoperative deep venous thrombosis, which may be as high as 62% in patients with delayed surgery (25). Older patients are also at high risk of thrombosis because of their physiological characteristics (27). About 90% of symptomatic pulmonary embolism cases originate from DVT (15) in the lower extremities, which seriously threatens the safety of patients, so it is necessary to detect DVT before operation. If preoperative DVT is not detected and managed promptly in elderly patients with femoral neck fractures, deep venous thrombosis of the lower extremities may become larger and fall off during blows and bedridden postoperatively, which may subsequently lead to PE (16), a serious postoperative complication after total hip arthroplasty (THA) and a common cause of postoperative death, accounting for between 0.1% and 0.5% of patients after THA (17). Therefore, prophylactic anticoagulation is often required to prevent the development of DVT in elderly patients with femoral neck fractures upon admission (13). However, if DVT is diagnosed at admission, the dose of anticoagulants needs to be adjusted in a timely manner (18). Therefore, despite the limitations of these diagnostic tools, all elderly patients with femoral neck fractures need to undergo lower extremities venography or ultrasonography. There is no convenient tool to diagnose DVTs of the lower extremities.

In this study, we calculated 4 significant risk factor coefficients by multiple logistic regression using the common and rapid laboratory results and related case data after admission, which has high specificity and clinical significance. Paik B et al.'s study suggests that right iliac artery compression of left iliac vein may lead to higher left lower limb DVT (19), and we found that patients with right femoral neck fractures were 2.4 times more likely to have lower extremity venous thrombosis than patients with left femoral neck fractures, possibly due to the presence of both trauma and compression in these patients, which needs to be validated in further studies.

Previous studies have shown that low hemoglobin level may be a risk factor for venous thrombosis of the lower extremities in patients with intertrochanteric femoral fractures (20, 25), and that anemia also increases the risk of venous thrombosis of the lower extremities in elderly patients with femoral neck fractures (21). Optimizing preoperative hemoglobin level reduces anemia and adverse outcomes after total joint replacement. However, some studies have pointed out that the preoperative and postoperative hemoglobin values are not related to the prognosis of patients (26). The possible explanation may be that we excluded factors affecting preoperative hemoglobin such as high-energy injury, open fracture and multiple fractures. Some studies have shown that postoperative fibrinogen, a glycoprotein synthesized in the liver and involved in coagulation, is associated with an increased risk of venous thrombosis (4, 22, 25). Fibrinogen promotes platelet aggregation and the growth, proliferation and contraction of smooth muscle and endothelial cells, increases blood viscosity and peripheral resistance, causes endothelial cell damage and thus accelerates thrombosis (23, 25). Therefore, monitoring the level of fibrinogen is of clinical significance for the diagnosis of cardiovascular diseases and the screening of thrombosis. Plasma d-dimer level is an important marker of fibrinolytic activity, reflecting high coagulation and fibrinolytic activity. A d-dimer of 500 ng/mL had a sensitivity of 90%–100% for the diagnosis of DVT, but the specificity is low (4). It is susceptible to a variety of factors, including age, inflammation, malignancy and post-traumatic stress (24). The optimal critical value for the d-dimer level was 2.4 mg/L, and the risk of DVT exceeding this value increased significantly and was not affected by multivariate variables. But its specificity of 36.1% was still low. While, the factor of d-dimer can be used as an auxiliary factor to improve diagnostic accuracy. Using the formula (new predictive index = 0.895 * injured side (right) + 0.899 * hemoglobin (<109.5 g/L) + 1.19 * fibrinogen (>4.24 g/L) + 1.221 *d-dimer (>2.4 mg/L.)) to calculate the DVT index, the sensitivity was 51.4%, the specificity was 89.8%, and the AUC value was 0.735.

Overall, we used a multicenter sample to analyze multiple possible risk factors with significant impact and to identify incidence and risk factors for DVT in elderly patients with traumatic femoral neck fractures, which will facilitate the clinical management of these patients. In addition, a formula derived from these risk factors can be used to assist in the preoperative diagnosis of femoral neck fractures and to identify the risk of DVTs quickly. For elderly patients with multiple high-risk factors, we recommend lower extremity ultrasound examination to early diagnose thrombus and timely anticoagulant treatment. Finally, the risk factors included in the final formula are clearly defined and easy to collect, and have certain practicability.

This study has several limitations. First, retrospective design has inherent limitations in the accuracy of data collection, and the number of cases included is small. Second, as with other multivariate analyses, we cannot include all confounding factors, and the remaining confounding is still a problem. Third, some key variables that are important to the development of DVT are not available, including BMI (body mass index). Fourth, the accuracy of ultrasonography in the diagnosis of DVT may be lower than that of venography. Therefore, further large-scale clinical studies and prospective studies are needed to strengthen our findings.

## Conclusions

In conclusion, our results suggested that in elderly patients with traumatic femoral neck fractures, the incidence of DVT was higher at admission based on ultrasound results, reaching 18.2%, which was consistent with previous studies. Our study also showed that independent risk factors such as right femoral neck fracture, anemia, high d-dimer and fibrinogen levels were associated with an increased risk of DVT in elderly patients with femoral neck fracture. Using multivariate logistic regression analysis, our study defined a new index for diagnosing DVT based on risk factors with the AUC value of 0.735. This index may be helpful for clinicians to diagnose venous thrombosis of lower limbs.

## Data Availability

The raw data supporting the conclusions of this article will be made available by the authors, without undue reservation.
